# The association between cognitive impairment and 30-day mortality among older Chinese inpatients

**DOI:** 10.3389/fmed.2022.896481

**Published:** 2022-08-24

**Authors:** Xiao-Ming Zhang, Jing Jiao, Na Guo, Chen Zhu, Zhen Li, Dongmei Lv, Hui Wang, Jingfen Jin, Xianxiu Wen, Shengxiu Zhao, Xinjuan Wu, Tao Xu

**Affiliations:** ^1^Department of Nursing, Peking Union Medical College Hospital (Dongdan Campus), Chinese Academy of Medical Sciences - Peking Union Medical College, Beijing, China; ^2^Department of Nursing, The Second Affiliated Hospital of Harbin Medical University, Harbin, China; ^3^Department of Nursing, Tongji Medical College, Tongji Hospital, Huazhong University of Science and Technology, Wuhan, China; ^4^Department of Nursing, The Second Affiliated Hospital Zhejiang University School of Medicine, Hangzhou, China; ^5^Department of Nursing, Sichuan Provincial People's Hospital, Chengdu, China; ^6^Department of Nursing, Qinghai Provincial People's Hospital, Xining, China; ^7^Department of Epidemiology and Statistics, Peking Union Medical College, Institute of Basic Medical Sciences, Chinese Academy of Medical Sciences and School of Basic Medicine, Beijing, China

**Keywords:** cognitive impairment, mortality, inpatients, older adults, ADL

## Abstract

**Purpose:**

Although the association between cognitive impairment and mortality has been widely described among community-dwelling older adults, this association in hospitalized patients was limited.

**Objectives:**

This study's purpose was to explore the association between cognitive impairment and 30-day mortality after adjustment of factors among Chinese in-patients.

**Methods:**

This was a large-scale prospective study based on a cohort of patients aged 65 years and older, whose cognitive function was assessed using the Mini-Cog instrument, followed up at 30-days for mortality. Multivariate logistic regression models were used to assess the association between cognitive impairment and 30-day mortality.

**Results:**

There were 9,194 inpatients in our study, with an average age of 72.41 ± 5.72. The prevalence of cognitive impairment using the Mini-Cog instrument was 20.5%. Multivariable analyses showed that patients with cognitive impairment have an increased risk of 30-day mortality, compared to those with normal cognitive function (OR = 2.83,95%CI:1.89–4.24) in an unadjusted model. In the fully adjusted model, Patients with cognitive impairment had an increased risk of 30-day mortality compared to those with normal cognitive function in the completely adjusted model (OR = 1.76,95% CI: 1.14–2.73). Additionally, this association still existed and was robust after performing a stratified analysis of age, gender, frailty and depression, with no significant interaction (*P* > 0.05).

**Conclusions:**

Our study found that older Chinese in-patients with cognitive impairment have a 1.76-fold risk of 30-day mortality compared to patients with normal cognitive function, suggesting that clinicians and nurses need to early implement cognitive function screening and corresponding interventions to improve clinical outcomes for older in-patients.

## Introduction

With advances in medicine and technology, the world's population is aging, a trend that is especially noticeable among the Chinese people, with an estimated 366 million older Chinese adults expected by 2050 (https://population.un.org/wpp/DataQuery). Elderly individuals often suffer from cognitive impairment, a disease characterized by declining memory and trouble learning new things, with the likelihood of progressing to dementia in the future (1). It has been reported that the prevalence of cognitive impairment ranges from 9.7% to 23.30% in Chinese people, with a figure of 20.8% for people aged 65 years and older (2). Numerous studies have reported that being an older adult with cognitive impairment is associated with a series of poor clinical outcomes, such as falls (3), lower quality of life (4), and increased mortality (5), resulting in a substantial healthcare burden for families and society alike. In mainland China, it is estimated that the annual healthcare cost for patients with Alzheimer's disease is $167.74 billion US, far more than the estimate reported by the World Alzheimer Report of $28.18 billion US (6).

Several studies have reported that community-dwelling older adults with cognitive impairment have an increased risk of mortality compared to those with normal cognition (7–10). A large cohort study recently reported that community-dwelling older adults with cognitive impairment are at increased risk of mortality compared to those with normal cognitive function (11). The possible reasons for the association between cognitive impairment and mortality were etiologic conditions such as multiple comorbidities (12) or inability to efficiently process information, resulting in noncompliance with medical care and unlikely maintenance of a healthier lifestyle (13). Moreover, the impact of cognitive impairment on adverse clinical outcomes among hospitalized older adults has become increasingly studied (14, 15). Indeed, inpatients suffering from cognitive impairment may have worse clinical outcomes than community-dwelling older adults due to forgetting to take medication or failing to follow clinicians' advice. In addition, poor functional capacity and multiple comorbidities make older inpatients more vulnerable to the detrimental effect of cognitive impairment than older people living in the community. Therefore, identifying the association between cognitive impairment and mortality among hospitalized older people is very important.

Several studies from Western countries have explored the impact of cognitive impairment on mortality among older adults in hospital settings (16–19). One study involving 539 emergency department inpatients reported that patients with cognitive impairment had an approximately 3.10-fold higher risk of mortality than those with normal cognitive function, indicating that emergency medical personnel need to pay much more attention to older inpatients (19). Similar results were also found in another study (18). However, these abovementioned studies focused on western countries. To date, one study has explored the association between cognitive or physical frailty and the composite outcome of readmission and death among Chinese elderly inpatients with cardiovascular disease, reporting that elderly inpatients with cognitive impairment were not associated with worse outcomes compared to robust patients (20). However, this study was from a single center with negative results. Therefore, a prospective multicenter study with a large-scale cohort sample exploring the association with mortality among older Chinese inpatients is needed. Our aim was to use a national prospective cohort study to estimate the prevalence of cognitive impairment in older inpatients and identify whether there was an independent association between cognitive impairment and 30-day mortality among older inpatients in China after adjusting for age, sex, education frailty, depression, and activities of daily living (ADL). Moreover, our study also examined the association between cognitive impairment and mortality with different subgroup analyses, such as sex, age group, depression, and frailty. We hypothesized that there is an independent association between cognitive impairment and 30-day mortality in older Chinese inpatients.

## Materials and methods

### Study design and participants

This prospective study was a large-scale cohort representing older Chinese inpatients in a national baseline survey with data from October 2018 to February 2019. A previous protocol was registered in the Chinese Clinical Trial Registry with the number ChiCTR1800017682 (21). Detailed information on the sampling methods was described in a previously published article (21). Overall, this study used a two-stage cluster sampling method. The Chinese administrative authority divided this country into six administrative regions determined by the level of the economy and varying geography. In the first stage, we randomly selected one province or municipality from each administrative region, and then one tertiary hospital was sampled at random. Inpatients coming from internal and surgical wards in the selected tertiary hospital were recruited. Subsequently, inpatients aged 65 years or older who gave their informed consent to participate in the study were included. Inpatients at the end stage of severe disease and those experiencing unconsciousness were excluded; inpatients with delirium or dementia that not measured for depression were also excluded. Investigators collected the information in face-to-face interviews.

### Cognitive impairment assessment

Baseline cognitive impairment was evaluated by the Chinese version of the Mini-Cog, which was tested in a Chinese population (22). We adopted the Mini-Cog screening instrument, a three-minute tool consisting of two components: a three-item recall test for memory and one simple scored clock (23). The total score was five points, and inpatients scoring ≤3 were defined as having cognitive impairment. This cognitive screening instrument has been widely applied in a clinical setting because it is quick, simple, and has a similar sensitivity to cognitive impairment as the Mini-Mental State Examination (MMSE) (24). All investigators were registered nurses and were educated and trained before conducting this investigative study.

### Assessment of baseline covariates

Demographic variables, including sex, age, education, ethnicity, and marital status, were obtained based on self-report. We classified the education category into four levels: illiterate, primary school, middle school, and university. Marital status (married, divorced, or widowed), alcohol consumption (current drinker, former drinker, or nondrinker), tobacco use (current smoker, former smoker, or nonsmoker), and ethnicity (Han or minority) were dichotomous variables. We also investigated other important covariates, including hearing, vision, alcohol and tobacco use, sleep quality, bedridden for an extended period of time, urinary function, depression, frailty, body mass index (BMI), and basic activities of daily living (ADL). Hearing and vision were defined as whether they affected normal life; additionally, urinary function and sleep were assessed as dysfunctional or normal.

Depression was evaluated using the Geriatric Depression Scale 15 (GDS15) score instrument (25), consisting of 15 questions to evaluate what inpatients felt. When the cutoff score was above 5, an inpatient was defined as having depression.

The FRAIL scale was used to assess frailty and is widely applied in different settings, ranging from the community (26) to hospitals (27) and nursing homes (28). This frailty score instrument consisted of five components: fatigue, resistance, ambulation, illness, and weight loss, with a cutoff ≥3 defining frailty (29).

We used the Barthel Index and Instrumental Activities of Daily Living to evaluate inpatient ADLs. This ADL index instrument consisted of 10 questions, with scores ranging from 0 to 100 (30). A higher ADL score meant better ADL function.

### Outcome

After the baseline survey, a trained nurse followed up on the clinical outcomes and mortality at 30 days by conducting telephone interviews using professional skills.

### Quality control

The survey quality was very important, and we scrutinized the quality by implementing several measures. First, every investigator needed to attend professional training to fully use all score instruments, including the Barthel Index, Instrumental Activities of Daily Living, GDS15, Mini-Cog, and FRAIL scale. Second, to maintain data accuracy and integration, two investigators needed to double-check every case report form to ensure the authenticity of the raw data. Third, we used an electronic data collection system to store the raw data, which allowed it to be exported correctly.

### Statistical analyses

Categorical variables and continuous variables are presented as percentages and means (±standard deviations), respectively. Student's *t*-test and the chi-square test were used for continuous variables and categorical variables, respectively, to detect differences between the two groups. Bivariate logistic regression analysis was used to explore the association between cognitive impairment and 30-day mortality. A different adjustment model was also listed: (1) unadjusted; (2) age, sex, and education adjusted;(3) age, sex, education, depression, frailty, and ADL adjusted. Additionally, we performed subgroup analysis based on sex and age group (65–75 years old vs. >75 years old) by stratified analysis. We used a two-tailed *P* < 0.05 as a significant difference. All statistical analysis methods were conducted using SAS 9.4 software (SAS Institute Inc., Cary, NC, USA).

## Results

### Patient characteristics

**Figure 1:**
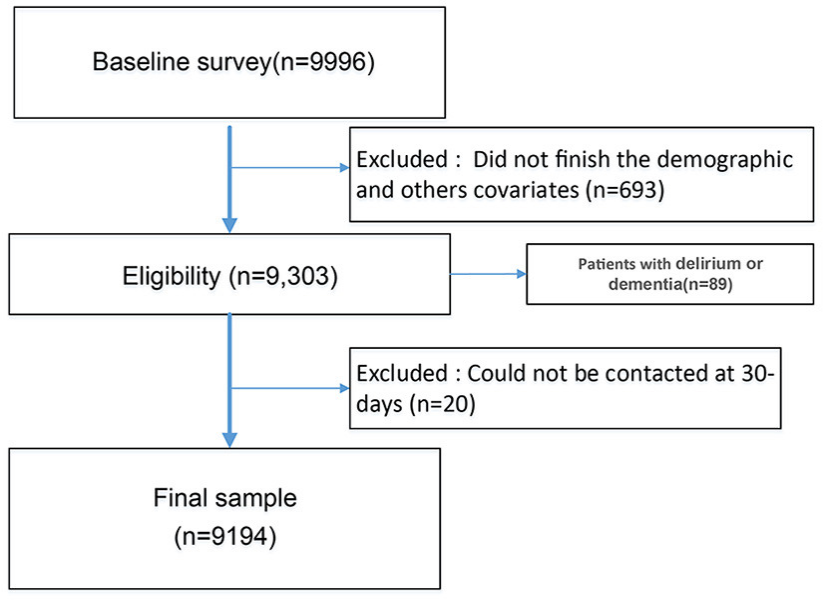
Flowchart of study participant.

At the initial stage, we recruited 9,996 patients, with 9,303 participants finishing the demographic and cognitive assessment questionnaire survey. And 89 patients did not finish GDS measurement due to delirium or dementia, leaving 9,194 subjects for the final analysis, with 20 participants lost to follow-up (Figure 1). Overall, this cohort's mean age was 72.41 (SD = 5.72), with 58.1% being male. A total of 20.5% (1,885/9,194) of patients were assessed as having cognitive impairment, of whom 15.5% were illiterate. Most patients (94.5%) were of Han nationality, and 89.0% were married. The percentages of frailty and depression were 16.9% and 16.2%, respectively.

### Subjects with normal cognition vs. subjects with cognitive impairment

The baseline characteristics and demographics between cognitive impairment and normal cognitive function are shown in Table 1. Overall, the 1,885 inpatients with cognitive impairment were older, predominantly female, had a higher prevalence of depression and frailty, had a higher incidence of being divorced or widowed, had a higher incidence of being bedridden, and had more vision, hearing, or sleep dysfunction compared with the 7,309 participants with normal cognitive function. In addition, inpatients with cognitive impairment had lower BMI and ADL than inpatients without cognitive impairment.

**Table 1:** Demographics characteristics of study participants [n (%)].

**Characteristics**	**Total** **(n = 9,194)**	**Subjects with** **normal cognition** **(7,309)**	**Subjects with** **cognitive impairment** **(1,885)**	***P value***
Age (mean, SD)	72.41 ± 5.72	72.07 ± 5.53	73.72 ± 6.25	<0.0001
ADL (mean, SD)	90.37 ± 17.9	91.60 ± 16.65	85.56 ± 21.78	<0.0001
BMI (mean, SD)	23.60 ± 3.49	23.70 ± 3.43	23.22 ± 3.72	<0.0001
Gender (n, %)				<0.0001
Female	3,851(41.9)	2871 (74.55)	980 (25.45)	
Male	5,343(58.1)	438 (83.06)	905 (16.94)	
Ethnicity (n, %)				
Han	8,685(94.5)	6,980 (80.37)	1,705 (19.63)	<0.0001
Other	509(5.5)	329 (64.64)	180 (35.36)	
Education (n, %)				<0.0001
Illiterate	1,430 (15.6)	836 (58.46)	594 (41.54)	
Primary school	2,636 (28.7)	1998 (75.80)	638 (24.20)	
Middle school	3,756 (40.9)	3236 (86.16)	520 (13.84)	
University	1,370 (14.9)	1,237 (90.29)	133 (9.71)	
Frailty (n, %)				<0.0001
Yes	1,551 (16.9)	1,053 (67.89)	498 (32.11)	
No	7,643 (83.1)	6,256 (81.85)	1,387 (18.15)	
Marital status (n, %)				<0.0001
Married	8,175 (89.0)	6,598 (80.71)	1,577 (19.29)	
Divorced or widowed	1,009 (11.0)	703 (69.67)	306 (30.33)	
Smoking status (n, %)				0.0003
Current smoker	1,027 (11.2)	838 (81.60)	189 (18.40)	
Former smoker	2,122 (23.1)	1,740 (82.0)	382 (18.0)	
Non-smoker	6,045 (65.7)	4,731 (78.26)	1,314 (21.74)	
Alcohol consumption (n, %)				<0.001
Current drinker	1,084 (11.8)	917 (84.59)	167 (15.41)	
Former drinker	1,110 (12.1)	903 (81.35)	207 (18.65)	
Non-drinker	7,000 (76.1)	5,489 (78.41)	1,511 (21.59)	
Bedridden (n, %)				<0.001
Yes	228 (2.5%)	150 (65.79)	78 (34.21)	
No	8,966 (97.5)	7,159 (79.85)	1,807 (20.15)	
Vision (n, %)				0.0001
Normal	7,318 (79.6)	5,877 (80.31)	1,441 (19.69)	
Dysfunction	1,876 (20.4)	1,432 (76.33)	444 (23.67)	
Hearing (n, %)				0.003
Normal	7,558 (82.2)	6,052 (80.07)	1,506 (19.93)	
Dysfunction	1,636 (17.8)	1,257 (76.83)	379 (23.17)	
Sleep (n, %)				<0.001
Normal	5,252 (57.1)	4,309 (82.04)	943 (17.96)	
Dysfunction	3,942 (42.9)	3,000 (76.10)	942 (23.90)	
Urinary function (n, %)				0.501
Normal	7,916 (86.1)	6,302 (79.61)	1,614 (20.39)	
Dysfunction	1,278 (13.9)	1,007 (78.79)	271 (21.21)	
Depression (n, %)				<0.001
Yes	1,487 (16.2)	1,036 (69.7)	451 (30.3)	
No	7,707 (83.8)	6,273 (81.4)	1,434 (18.6)	

### Univariate analysis between survivors and deceased patients at 30 days

Deceased patients were more likely to be older than inpatients who survived (*P* = 0.015). In addition, compared with survivors, deceased patients had a higher prevalence of depression (*P* < 0.001), frailty (*P* < 0.001), cognitive impairment (*P* < 0.001), being bedridden for an extended period (*P* = 0.0003), and sleep dysfunction. Deceased patients had significantly lower ADL and BMI values than survivors. However, differences in sex, ethnicity, marital status, smoking status, alcohol use, hearing, and urinary function between these two groups were not statistically significant (*P* > 0.05), as shown in Table 2.

**Table 2:** Univariate analysis results.

**Characteristics**	**Survivors at 30-day (*n* = 9,096)**	**Deceased at 30-day (*n* = 98)**	***P* value**
Age (mean, SD)	72.39 ± 5.7	73.79 ± 6.15	0.015
ADL (mean, SD)	90.56 ± 17.74	72.09 ± 28.52	<0.001
BMI (mean, SD)	23.62 ± 3.49	22.02 ± 3.90	<0.001
Cognitive impairment (n, %)			<0.001
Yes	1,844 (97.82)	41 (2.18)	
No	7,252 (99.22)	57 (0.78)	
Gender (n, %)			0.829
Female	3,811 (98.96)	40 (1.04)	
Male	5,285 (98.91)	58 (1.09)	
Ethnicity (n, %)			0.252
Han	8,595 (98.96)	90 (1.04)	
Other	501 (98.43)	8 (1.57)	
Education (n, %)			0.0001
Illiterate	1,409 (98.53)	21 (1.47)	
Primary school	2,592 (98.33)	44 (1.67)	
Middle school	3,728 (99.25)	28 (0.75)	
University	1,365 (99.64)	5 (0.36)	
Frailty (n, %)			<0.001
Yes	1,507 (97.16)	44 (2.84)	
No	7,589 (99.29)	54 (0.71)	
Marital status (n, %)			0.688
Married	8,089 (98.95)	86 (1.05)	
Divorced or widowed	997 (98.81)	12 (1.19)	
Smoking status (n, %)			0.175
Current smoker	1,011 (98.44)	16 (1.56)	
Former smoker	2,097 (98.82)	25 (1.18)	
Non-smoker	5,988 (99.06)	57 (0.94)	
Alcohol consumption (n, %)			0.429
Current drinker	1,073 (98.99)	11 (1.01)	
Former drinker	1,094 (98.56)	16 (1.44)	
Non-drinker	6,929 (98.99)	71 (1.01)	
Bedridden (n, %)			0.0003
Yes	220 (96.49)	8 (3.51)	
No	8,876 (99.00)	90 (1.00)	
Vision (n, %)			0.043
Normal	7,232 (98.82)	86 (1.18)	
Dysfunction	1,864 (99.36)	12 (0.64)	
Hearing (n, %)			0.881
Normal	7,478 (98.94)	80 (1.06)	
Dysfunction	1,618 (98.90)	18 (1.10)	
Sleep (n, %)			0.013
Normal	5,208 (99.16)	44 (0.84)	
Dysfunction	3,880 (98.63)	54 (1.37)	
Urinary function (n, %)			0.321
Normal	7,835 (98.98)	81 (1.02)	
Dysfunction	1,261 (98.67)	17 (1.33)	
Depression (n, %)			<0.001
Yes	1,455 (97.85)	32 (2.15)	
No	7,641 (99.14)	66 (0.86)	

### Evaluating the independent association between cognitive impairment and 30-day mortality

The results indicated that inpatients with cognitive impairment have an increased risk of 30-day mortality than those with normal cognitive function (Crude OR = 2.83, 95% CI: 1.89–4.24). In addition, this association still existed when adjusting for demographic variables such as age, sex, and education (OR= 2.30, 95% CI: 1.50–3.52). Finally, after adjusting for demographics and geriatric syndrome variables such as age, sex, education, frailty, depression, and ADL, cognitive impairment was still an independent risk factor for mortality (OR = 1.76, 95% CI: 1.14–2.73), as shown in Table 3.

**Table 3:** Multivariate logistic regression analysis of relationship between cognitive impairment and 30-day mortality in different models.

**Exposure**	**Non-adjusted** **(OR,95%, CI)**	**Adjusted I** **(OR,95%, CI)**	**Adjusted II** **(OR,95%, CI,)**	**Adjusted III** **(OR,95%, CI)**	**Adjusted IV** **(OR,95%, CI)**
Cognitive impairment					
No	Reference	Reference	Reference	Reference	Reference
Yes	2.83 (1.89–4.24)	2.30 (1.50–3.52)	2.00 (1.30–3.08)	1.96 (1.27–3.02)	1.76 (1.14–2.73)

Adjusted I: age gender education.

Adjusted II: age gender education, frailty.

Adjusted III: age gender education, frailty, depression.

Adjusted IV: age gender education, frailty, depression, ADL.

### Subgroup analysis by stratified methods

**Figure 2:**
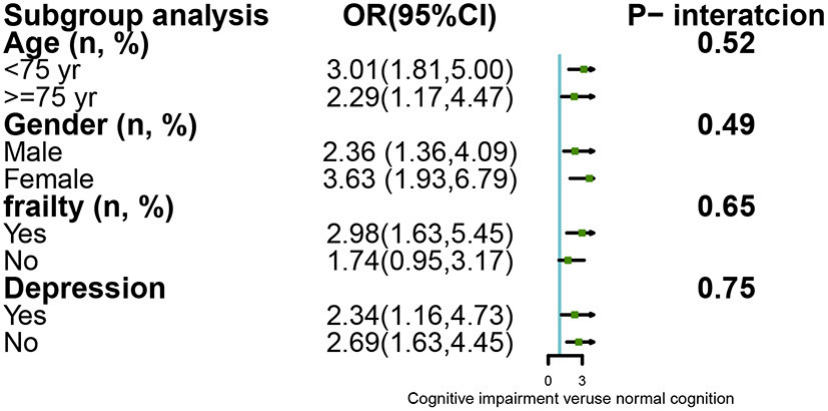
Subgroup analysis for the association between cognitive impairment and mortality by stratified and interaction analysis.

We explored the association between cognitive impairment and 30-day mortality by stratified analysis in terms of sex, age, frailty, and depression, with detailed information shown in Figure 2. The stratified analysis indicated that these associations between cognitive impairment and 30-day mortality were unchanged, with no significant interaction effect (*P* > 0.05).

## Discussion

To the best of our knowledge, this is the first study to explore the association between cognitive impairment and 30-day mortality among older Chinese inpatients with a large multicenter sample. Our study found that inpatients with cognitive impairment have an independent 1.76-fold increased risk of 30-day mortality compared to those without cognitive impairment after adjusting for demographics and geriatric syndrome variables. This indicates that early screening for cognitive function and participation in a corresponding intervention program by older hospitalized Chinese patients is urgently required to improve poor clinical outcomes.

In our large-scale cohort study, the prevalence of cognitive impairment was 20.5%, as assessed by the Mini-Cog assessment instrument. This result is higher than the 6.54% in an urban population and 7.43% in a rural population reported by a previous study using the MMSE score (31), but it is similar to other results (27%) (18). On the other hand, another study reported that the prevalence of cognitive impairment among older adults in an emergency surgical setting was 61.0% using Montreal Cognitive Assessment, with a cutoff score of ≤ 23 (19). The main reasons for these differences are subject age, disease type and condition, and different cognitive assessment tools. In general, hospitalized patients and older adults have a higher prevalence of cognitive impairments than community-dwelling older adults because comorbidity and greater disease severity are risks of cognitive impairment (32). However, screening and diagnosis for cognitive function in Chinese inpatients are limited. According to the results of a recent study (2), most patients with cognitive impairment are not diagnosed because of a lack of dementia specialists. This results in a large healthcare issue for those groups and prevents meeting the needs of people with dementia.

Several factors are known to determine the association between cognitive impairment and 30-day mortality. First, inpatients with cognitive impairment are at increased risk of mortality through etiologic conditions, indicating that patients with cognitive impairment suffer more from multiple diseases such as stroke, chronic obstructive pulmonary disease, and other multisystem disorders than patients with normal cognition (12). Second, cognitive impairment damages the capacity to obtain, understand, and even process medical services. This leads to poor health behaviors and an unhealthy lifestyle (33), including being unable to recognize the signs of disease, failing to follow prescribed medication regimes, and difficultly participating in regular exercise (13). Third, sarcopenia and frailty, two well-known geriatric syndromes, were reported as risk factors for cognitive impairment, as confirmed in a systematic review and meta-analysis (34, 35). Therefore, cognitive impairment, to a certain extent, reflects these two geriatric syndromes, which are associated with mortality. Although stratified analysis has shown that the association between cognitive impairment and 30-day mortality is higher in females (OR = 3.63; 95% CI: 1.93–6.79) than in males (OR = 2.36; 95% CI: 1.36–4.09), the interaction was not significant. Examining the sex-specific differences in the impacts of cognitive impairment on mortality, we were unable to draw conclusions based on previous studies. On the one hand, a previous study (36) described that the risk of mortality was higher in males than in females, possibly due to males' greater likelihood of smoking and drinking, whereas others (37) reported that females demonstrate a higher risk of mortality. We believe the main reasons might be due to different ethnicities, regions, sample sizes, and education levels (11). Therefore, more evidence is required to explore this important issue.

Our study found that inpatients with cognitive impairment have an increased risk of 30-day mortality than inpatients with normal cognitive function. This association still existed after performing a stratified analysis, consistent with previous studies reporting this association in different types of older patients (7, 9, 38–41). In recent years, the impact of cognitive impairment on mortality in Chinese community-dwelling older adults (11, 36) has been well studied; however, few studies have been conducted on hospitalized patients (18). The prevalence of mortality in inpatients was higher than that in community-dwelling older adults, and the adverse impact of cognitive impairment on clinical outcomes was also severe in hospitals, with low levels of recognition by medical personnel. Our study implies that early screening and diagnosis for cognitive impairment is very significant. Once patients are screened for cognitive impairment in hospital wards, corresponding interventions need to be carried out. For example, clinicians can write a comprehensive prescription, such as psychosocial interventions (42), cognitive therapy (cognitive stimulation therapy, cognitive rehabilitation, cognitive training) (43), and medications for patients. Medical health education interventions also need to be implemented for both patients and their family members. Thus, the more often and earlier the cognitive impairment screening is performed, the better the clinical outcome will be.

There are several strengths and limitations in our study. First, our study included a large-scale sample size and was a multicenter study that explored the association between cognitive impairment and 30-day mortality in older Chinese inpatients. Second, we used different adjusted models and sensitivity analyses, suggesting that our results are robust. However, some limitations need to be considered. First, the follow-up in our larger-scale cohort study is relatively short compared with other published studies (one-year mortality or more), which fails to generalize the association of long-term cognitive impairment with mortality. Second, the Mini-Cog cognitive function assessment tool is a screening tool and cannot truly diagnose, which may underestimate or overestimate the prevalence of cognitive impairment and further influence the impact of cognitive impairment on 30-day mortality. Third, although we adjusted for some factors related to 30-day mortality, such as depression, ADL, and frailty, we did not control for other factors, comorbidities, or potential confounding factors, possibly influencing the reported results. To address this issue, we are currently continuing the multiple-center follow-up study for a 1-year period. Furthermore, future prospective cohort studies exploring the association between cognitive impairment and mortality, adjusting for more potential confounding factors, among older Chinese inpatients with a long-term follow-up period should be implemented, and a larger samples of randomized controlled trials should be implemented to identify whether effective interventions for cognitive impairment could help to reduce the rate of short-term or long-term mortality.

## Conclusions

In this large multicenter longitudinal study, cognitive impairment was prevalent in older Chinese hospitalized patients and was significantly associated with an increased risk of 30-day mortality. These observations are relevant to clinicians and nurses because they can potentially identify high-risk older inpatients for poor clinical outcomes. As a result, corresponding adjustment interventions, including early geriatric involvement and cognitive therapy, combined with a family program, can be preemptively performed. We suggest that clinical medical personnel routinely perform early cognitive function screening with bedside tools on admission to carry out a comprehensive therapeutic regimen for older inpatients.

## Data availability statement

The raw data supporting the conclusions of this article will be made available by the authors, without undue reservation.

## Ethics statement

The studies involving human participants were reviewed and approved by the Ethics Committee of Peking Union Medical College Hospital (S-K540). The patients/participants provided their written informed consent to participate in this study.

## Author contributions

XWu responsible for the concept and design. TX analyzed and interpreted the data. X-MZ and JJia participated in designing the study and drafted the initial manuscript. NG, CZ, ZL, DL, HW, JJin, XWen, and SZ carried out the data acquisition and patient interviews. All authors contributed to the article and approved the submitted version.

## Funding

This work was supported by the Special Research Fund for Central Universities, Research Centre of Nursing Theory and Practice, Peking Union Medical College [Grant Number 2018PT33001] and CAMS Innovation Fund for Medical Sciences [Grant Number 2018-I2M-AI-009].

## Conflict of interest

The authors declare that the research was conducted in the absence of any commercial or financial relationships that could be construed as a potential conflict of interest.

## Publisher's note

All claims expressed in this article are solely those of the authors and do not necessarily represent those of their affiliated organizations, or those of the publisher, the editors and the reviewers. Any product that may be evaluated in this article, or claim that may be made by its manufacturer, is not guaranteed or endorsed by the publisher.
